# Dysfunction of Endothelial Progenitor Cells from Smokers and Chronic Obstructive Pulmonary Disease Patients Due to Increased DNA Damage and Senescence

**DOI:** 10.1002/stem.1488

**Published:** 2013-12-03

**Authors:** Koralia E Paschalaki, Richard D Starke, Yanhua HU, Nicolas Mercado, Andriana Margariti, Vassilis G Gorgoulis, Anna M Randi, Peter J Barnes

**Affiliations:** a Airway Disease Section and National Heart and Lung Institute, Faculty of Medicine, Imperial College LondonLondon, United Kingdom; b Vascular Sciences, National Heart and Lung Institute, Faculty of Medicine, Imperial College LondonLondon, United Kingdom; c Histology-Embryology Department, Faculty of Medicine, University of AthensAthens, Greece; d Cardiovascular Division, King's College London British Heart Foundation CentreLondon, United Kingdom; e Biomedical Research Foundation, Academy of AthensAthens, Greece

**Keywords:** Endothelial progenitor cells, Smoking, DNA damage response, Sirtuin, Cellular senescence, Ataxia telangiectasia-mutated kinase

## Abstract

Cardiovascular disease (CVD) is a major cause of death in smokers, particularly in those with chronic obstructive pulmonary disease (COPD). Circulating endothelial progenitor cells (EPC) are required for endothelial homeostasis, and their dysfunction contributes to CVD. To investigate EPC dysfunction in smokers, we isolated and expanded blood outgrowth endothelial cells (BOEC) from peripheral blood samples from healthy nonsmokers, healthy smokers, and COPD patients. BOEC from smokers and COPD patients showed increased DNA double-strand breaks and senescence compared to nonsmokers. Senescence negatively correlated with the expression and activity of sirtuin-1 (SIRT1), a protein deacetylase that protects against DNA damage and cellular senescence. Inhibition of DNA damage response by silencing of ataxia telangiectasia mutated (ATM) kinase resulted in upregulation of SIRT1 expression and decreased senescence. Treatment of BOEC from COPD patients with the SIRT1 activator resveratrol or an ATM inhibitor (KU-55933) also rescued the senescent phenotype. Using an *in vivo* mouse model of angiogenesis, we demonstrated that senescent BOEC from COPD patients are dysfunctional, displaying impaired angiogenic ability and increased apoptosis compared to cells from healthy nonsmokers. Therefore, this study identifies epigenetic regulation of DNA damage and senescence as pathogenetic mechanisms linked to endothelial progenitors' dysfunction in smokers and COPD patients. These defects may contribute to vascular disease and cardiovascular events in smokers and could therefore constitute therapeutic targets for intervention.

## Introduction

Endothelial progenitor cells (EPC) are circulating stem cells that have the ability to differentiate into mature endothelial cells, thereby contributing to postnatal vasculogenesis and endothelial repair at sites of endothelial damage 1,2. Blood outgrowth endothelial cells (BOEC, also called endothelial colony forming cells [ECFC]), are a well-characterized endothelial cell population with robust clonal proliferative potential and ability to form *de novo* vessels *in vivo* 3. This population has recently attracted considerable interest as a potential cell-based therapy for vascular regeneration 4–6, gene therapy 7,8, and as a tool to study endothelial dysfunction in patients 9–12. Endothelial progenitors contribute to vascular homeostasis, thus their reduction or dysfunction could be involved in the development of endothelial dysfunction and cardiovascular disease (CVD) 13–17.

Cigarette smoke-oxidative stress is a major risk factor for CVD 18 and also the main risk factor for the development of chronic obstructive pulmonary disease (COPD), an obstructive lung inflammatory disorder affecting approximately 20% of smokers 19. Smokers with COPD are more likely to develop CVD than smokers with normal lung function 20, and CVD is the leading cause of death in COPD 21. Numerous studies have described endothelial dysfunction in young healthy smokers and in COPD patients 22,23 and have suggested that reduced numbers and dysfunction of EPC could contribute to CVD in these groups 24–29. However, the molecular process that links smoking and COPD with CVD is still unclear.

DNA damage pathways are important contributors to aging disorders, including COPD and CVD 30–34. DNA damage, caused by factors such as oxidative stress, activates ataxia telangiectasia mutated (ATM) kinase, a key player in the DNA damage response (DDR) 35,36; this can result in cell cycle arrest, senescence, or apoptosis. Recent evidence shows increased DNA damage and senescence in lung biopsies from smokers and COPD patients 34,37, which may contribute to accelerated lung aging and pathogenesis of COPD 34,38,39. Increased DNA damage and senescence is also evident in atherosclerotic plaques 33,40. Senescent vascular cells exhibit dysfunctional characteristics 33,41,42 and have been shown to contribute to accelerated vascular aging and atherosclerosis 43. Recent studies suggest that accumulation of DNA damage in stem and tissue specific progenitor cells can result in senescence and loss of their self-renewal ability, tissue aging, and/or stem cell depletion 44–47. Therefore, increased DNA damage is currently considered a causative link in the development of COPD, CVD, and tissue-specific stem cells aging.

An important regulator of genomic stability and cellular senescence is Sirtuin-1 (SIRT1), a nicotinamide adenine dinucleotide (NAD+)-dependent class III protein deacetylase 48. SIRT1 is recruited to sites of DNA double-strand breaks (DSB) induced by oxidative stress and is required for their efficient repair and maintenance of genomic stability 49. Cigarette smoke-oxidative stress has been shown to reduce SIRT1 levels not only *in vitro* but also in lung tissue from smokers and COPD patients 50,51. SIRT1 also inhibits endothelial senescence and appears to have a prominent protective role in vascular cells 52,53. For these reasons, SIRT1 is currently considered an important therapeutic target for age-related disorders, including COPD and CVD 54–58.

The purpose of this study was to investigate whether EPC are dysfunctional in smoking individuals and patients with COPD due to increased DNA damage imposed by cigarette smoke, which could contribute to the development of CVD and to elucidate the pathways involved in this process.

## Materials and Methods

### Participants

Blood samples (15–50 mL) were collected from healthy nonsmoking volunteers, smokers with normal lung function (forced vital capacity in 1 second (FEV_1_) >80% predicted, FEV_1_/forced vital capacity (FVC) >0.7), and COPD patients (FEV_1_<80% predicted, FEV_1_/FVC <0.7). All individuals (aged 38–80 years) were free from significant cardiac, renal, hematological, or other major disorders as determined by history, physical examination, and screening investigations. All COPD patients were current or ex-smokers and were classified according to the Global initiative for chronic Obstructive Lung Disease (GOLD) criteria for severity of disease 19. All volunteers were stable (no chest or other infection requiring antibiotics and/or oral steroids) for at least 4 weeks. The study was approved by the Royal Marsden, Hammersmith and Queen Charlotte's Ethics Committees, and informed consent was obtained from all individuals.

### Isolation and Culture of BOEC from Peripheral Blood

Peripheral blood mononuclear cells were isolated from blood samples and seeded at a density of 3–5 × 10^7^ cells per well, in complete endothelial growth medium (EGM)−2 (Lonza, Walkersville, MD, http://www.lonza.com), supplemented with 10% fetal bovine serum (FBS) (Hyclone, Thermo Scientific, Fisher Scientific Ltd., Loughborough, U.K., http://www.hyclone.com), onto six-well plates precoated with type I rat tail collagen (BD Biosciences, Bedford, MA, http://www.bdbiosciences.com), as previously described 11,59. After 24 hours, nonadherent cells and debris were aspirated, adherent cells were washed once with EGM-2 medium, and fresh EGM-2 was added to each well. Medium was changed daily for 7 days and then every 2 days. Colonies of BOEC appeared between 7 and 22 days in culture as discrete colonies of cells with cobblestone morphology and were enumerated by visual inspection using a ×4 objective lens (Olympus microscope CKX41 and Olympus microscope digital camera DP12-2, Olympus UK Ltd., London, U.K., http://www.olympus-global.com). Endothelial cells derived from the colonies were passaged for 2–3 weeks after appearance and grown to confluence.

### In Vivo Matrigel Plug Assay

BOEC from nonsmokers and COPD patients were labeled with the Vybrant DiI Cell-Labeling Solution (V-22885, Invitrogen Ltd., Paisley, U.K., http://www.invitrogen.com), according to the manufacturer's protocol. BOEC (5 × 10^5^) were mixed with 400 μL of Matrigel (BD Biosciences, 354234) and injected subcutaneously into the back or flank of NOD.CB17-Prkdcscid/NcrCrl mice. Seven days later, the mice were sacrificed and the plugs were harvested, frozen in liquid nitrogen, and cryosectioned. Experiments were performed for each BOEC sample in duplicate. Frozen sections were either fixed with 4% paraformaldehyde in PBS at 4°C overnight, and stained with hematoxylin and eosin stain (H&E) or fixed with acetone at 4°C for 10 minutes and analyzed by immunofluorescence confocal microscopy. Images for H&E staining were taken with a BX50 camera (Olympus) with Viewfinder software Version 3.0.1 (Pixera) using a ×10 and ×20 objective lenses. Images for immunofluorescence were captured using a Zeiss LSM510 META confocal microscope (Carl Zeiss, Welwyn Garden City, U.K., http://www.zeiss.com), using ×20, ×40, and ×63 objective lenses and running version 3.2 of the LSM acquisition software. At least three to five images were analyzed per sample. Volocity software (Improvision, Coventry, U.K., http://www.improvision.com) was used for quantification of cellular infiltration (H&E staining) and of Vybrant positive cells.

### Senescence Induced by Oxidative Stress

A modified method of a previously described protocol 53 for inducing premature senescence by H_2_O_2_ was used. Human umbilical vein endothelial cells (HUVEC) or BOEC 1 × 10^5^ were seeded in six-well plates and grown to 80% confluence in M199 medium (Sigma-Aldrich Company Ltd., Dorset, U.K., http://www.sigmaaldrich.com) plus 10% FBS. Cells were washed twice with PBS and treated for 1 hour with 25 μmol/L or 50 μmol/L of H_2_O_2_ (Sigma-Aldrich Company Ltd.). Cells were washed twice with PBS and cultured in M199 plus 10% FBS medium for two additional days.

### RNA Interference

RNA interference of ATM and SIRT1 expression was induced by small interference RNA (siRNA) from Dharmacon (ABgene Ltd., Surrey, U.K., http://www.abgene.com). Two siRNAs specific for ATM with different sequences (siGENOME SMART-pool Human ATM, M-003201-04-0005 and ON-TARGET plus Human ATM, J-003201-14-0010), a siRNA specific for SIRT1 (ON-TARGET plus SMART-pool Human SIRT1, L-03540-00-0005), and two control siRNAs (siGENOME Non-Targeting siRNA Pool, D-001206-14-05 and ON-TARGET plus Non-targeting siRNA, D-001810-01-05) were used. Transfection of BOEC or HUVEC was performed as previously described 11.

### Immunofluorescence

Isolated BOEC or HUVEC were stained as previously described 11 with antibodies to platelet endothelial cell adhesion molecule (PECAM) (CD31), vascular endothelial growth factor receptor (VEGFR)−2 (Abcam, Cambridge, U.K., http://www.abcam.com), vascular endothelial (VE)-cadherin (CD144) (BD Bioscience Pharmingen Oxford, U.K., http://www.bdbiosciences.com/), von Willebrand factor (VWF) (Dako UK Ltd., Cambridge, U.K., http://www.dako.com), CD45 (AbD Serotec, Oxford, U.K.), CD3 (Caltag Laboratories, Buckingham, U.K.), p16 and p21 (Santa Cruz Biotechnology, Insight Biotechnology Ltd., Wembley, U.K., http://www.scbt.com), 53 binding protein 1 (53BP1) and γ-H2AX (Cell Signaling Technology, New England Biolabs, Hertfordshire, U.K., http://www.cellsignal.com). Secondary antibodies were anti-mouse AlexaFluor 555 and anti-rabbit AlexaFluor 488 (Invitrogen Ltd.). Nuclei were visualized using TOPRO-3 (Invitrogen Ltd.).

Frozen sections of Matrigel plugs were stained with antibodies to CD31 (Abcam), p16 (Santa Cruz Biotechnology), 53BP1, and cleaved caspase-3 (Cell Signaling Technology). Nuclei were visualized using TOPRO-3 (Invitrogen Ltd.) or Draq-5 (Biostatus Limited, Leicester, U.K.). Terminal deoxynucleotidyl transferase dUTP nick end labeling (TUNEL) staining was performed with “In Situ Cell Death Detection Kit, Fluorescein” (Roche Diagnostics Ltd., West Sussex, U.K., http://www.roche-applied-science.com). Images were captured by confocal microscopy as described above.

### Senescence-Associated β-Galactosidase Staining

Senescence-associated β-galactosidase (SA-β-Gal) activity was measured with a β-Galactosidase staining kit (Senescence Detection Kit, BioVision Research Products, Mountain View, CA, http://www.bioscience.co.uk) following the manufacturer's protocol. The number of blue (senescent) cells relative to the total cell number was counted in two to four different optic fields, using ×10 or ×20 objective lens. At least 200 cells were counted per sample.

### SIRT1 Activity Assay

Nuclear extracts were prepared using a nuclear extraction kit (Active Motif Europe, Rixensart, Belgium, http://www.activemotif.com). SIRT1 activity was measured using a SIRT1 fluorescent activity assay kit (Fluor-de-Lys SIRT1 fluorometric drug discovery assay kit, Enzo Life Sciences, Exeter, U.K., http://www.enzolifesciences.com). Total SIRT1 activity and in the presence of a sirtuin inhibitor (nicotinamide) were determined. SIRT1 activity was calculated by subtracting nonspecific activity (activity in the presence of nicotinamide) from the total SIRT1 activity and expressed as μmol/L standard equivalent per μg protein.

### Western Blotting

Western blotting was carried out as described 11. The following antibodies were used: SIRT1 (Cell Signaling Technology), ATM, p21 (Santa Cruz Biotechnology), Glyceraldehyde 3-phosphate dehydrogenase (GAPDH) (Millipore, Watford, U.K., http://www.millipore.com), α-tubulin (Sigma-Aldrich Company Ltd.). Quantification of protein levels was performed by densitometry and normalized against GAPDH or α-tubulin.

### Real-Time Polymerase Chain Reaction

RNA was extracted from HUVEC treated with siRNA for ATM or control siRNA for 48 hours, as described 11. Levels of SIRT1 normalized to GAPDH mRNA were measured by quantitative real-time polymerase chain reaction (RT-PCR) using the following primers to SIRT1 (forward primer: 5′-CGTCTTATCCTCTAGTTCTTGTG-3′, reverse primer: 5′-ATCTCCATCAGTCCCAAATCC-3′) and GAPDH (forward primer: 5′-CAAGGTCATCCATGACAACTTTG-3′, reverse primer: 5′-GGGCCATCCACAGTCTTCTG-3′).

### Statistical Analysis

Data are expressed as means ± SEM. Statistical analysis was performed with SPSS Version 20 (IBM, Armonk, NY). Comparisons between multiple groups were performed using one-way ANOVA followed by a Fisher's least-significant difference post hoc test for normally distributed data or Games-Howell test for non-normally distributed data. Single comparisons were made with Student's *t* tests for normally distributed data or the Mann–Whitney *U* test for data not normally distributed. The correlation of values was estimated with the Spearman *r* correlation coefficient. Significance was defined as a *p* value of <.05.

## Results

### Isolation and Characterization of BOEC

**Figure 1:**
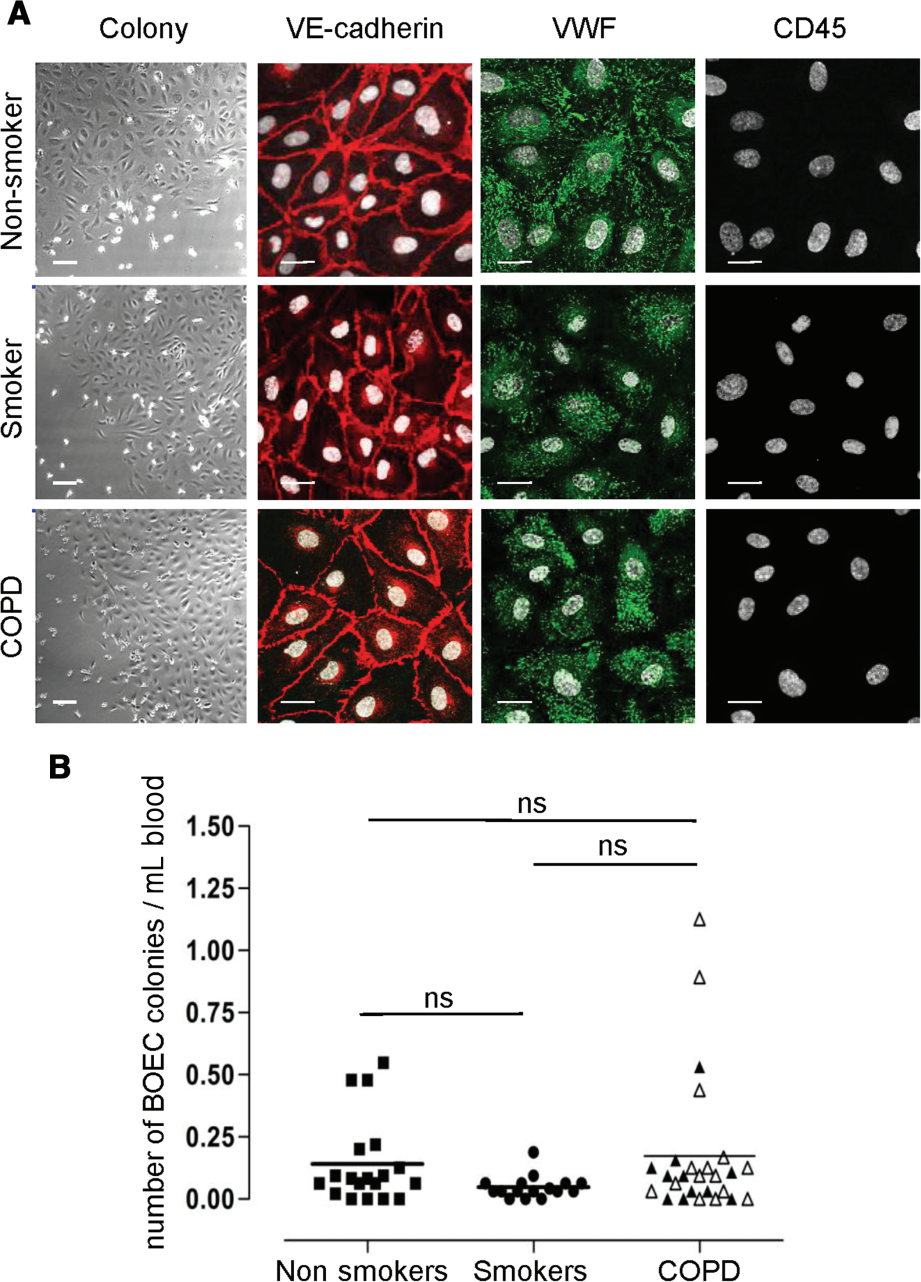
BOEC isolation and characterization. (A): Representative images of BOEC colonies in culture derived from healthy nonsmokers, healthy smokers, and COPD patients. Immunofluorescence (IF) staining for VE-cadherin (red), VWF (green), and CD45 (red). TOPRO-3 (white) was used as nuclear stain. BOEC from all groups were positive for the endothelial markers VE-cadherin and VWF and negative for the leukocytic CD45 marker (bright-field images scale bars = 100 μm; IF images scale bars = 20 μm). (B): Number of BOEC colonies in healthy nonsmokers, healthy smokers, and patients with COPD. The number of discrete BOEC colonies that appeared in the peripheral blood mononuclear cell culture was counted relative to the number of mL of blood obtained from healthy nonsmokers (*n* = 18), healthy smokers (*n* = 11), and COPD patients (*n* = 20) (open triangle: ex-smokers, black triangle: current smokers). Abbreviations: BOEC, blood outgrowth endothelial cells; COPD, chronic obstructive pulmonary disease; VWF, von Willebrand factor; VE, vascular endothelial.

Eighteen healthy nonsmokers, 11 smokers with normal lung function, and 20 COPD patients were enrolled in the study (for characteristics see Table1). Cultures from all groups, between day 7 and 22, gave rise to colonies of high proliferative potential, low proliferative potential, or no colonies at all (Fig. 1A, 1B; Supporting Information Fig. 1A–1C). Colonies appeared later in culture in healthy smokers compared to healthy nonsmokers (Supporting Information Fig. 1D). There was no difference in the number of BOEC colonies per milliliter of blood between the three groups (Fig. 1B; Supporting Information Fig. 1E). In two nonsmokers, five smokers and six COPD patients BOEC isolation was performed twice, since the first isolation procedure was unsuccessful. One healthy nonsmoker was excluded from the analysis due to a reduced white blood cell count of unknown etiology.

**Table 1:** Clinical characteristics of volunteers

Characteristic	Healthy nonsmokers	Healthy smokers	COPD
Number (*n*)	18	11	20 (3 mild, 12 moderate, 5 severe)^a^
Sex (M/F)	6/12	6/5	11/9
Age (years)	59 ± 2	58 ± 3	65 ± 2
Smoking (pack-years)	0	35 ± 7	51 ± 4 (10 ex-smokers)
Lung function			
FEV_1_, % predicted	97.6 ± 3.6	91.2 ± 2.1	62.1± 3.5^b^
FEV_1_/FVC, % predicted	75.9 ± 1.4	73.7 ± 2.0	53.8± 2.7^b^
Medication			
Statins	3	2	3
Inhaled steroids	0	0	8
CVD comorbidities			
Hypertension	2	0	1
Diabetes	0	0	1 (type 2)

Values are expressed as means ± SEM.

a Staging of COPD is according to the Global initiative for chronic Obstructive Lung Disease (GOLD) criteria.

b *p* < .001 (comparison between nonsmokers/smokers and COPD).

COPD, chronic obstructive pulmonary disease; FEV_1_, forced expiratory volume in 1 second; FVC, forced vital capacity; FEV_1_ and FEV_1_/FVC ratio are postbronchodilator for subjects with COPD, smokers or nonsmokers; F, female; M, male; pack-years, number of packs cigarettes smoked per day multiplied by the number of years of smoking.

Colonies were expanded and gave rise to morphologically homogeneous cultures (Supporting Information Fig. 1A–1C). There was no difference in the number of days that the colonies grew in culture before the first passage between the three groups (Supporting Information Fig. 1F, 1G). BOEC from colonies of high proliferative potential (expanded after passage 3) were obtained from 11 out of 18 healthy nonsmoking, 7 out of 11 healthy smoking, and 14 out of 20 COPD volunteers and were used for experiments between passage 3 and 6. The number of days between passage 2 and 3 (days ± SEM) was: 2.5 ± 0.2 in healthy nonsmokers, 4.6 ± 1.1 in healthy smokers, and 4.7 ± 1 in COPD patients, indicating a slower growth rate in healthy smokers and in COPD patients (even though significant only in healthy smokers) compared to nonsmokers.

The endothelial lineage of the cells was confirmed as described 11. All samples were positive for a range of endothelial cell markers including VE-cadherin, VWF, VEGFR-2, and CD31 and were negative for the lymphocytic and leukocytic markers CD3 and CD45 respectively (Fig. 1A; Supporting Information Fig. 2A–2C).

**Figure 2:**
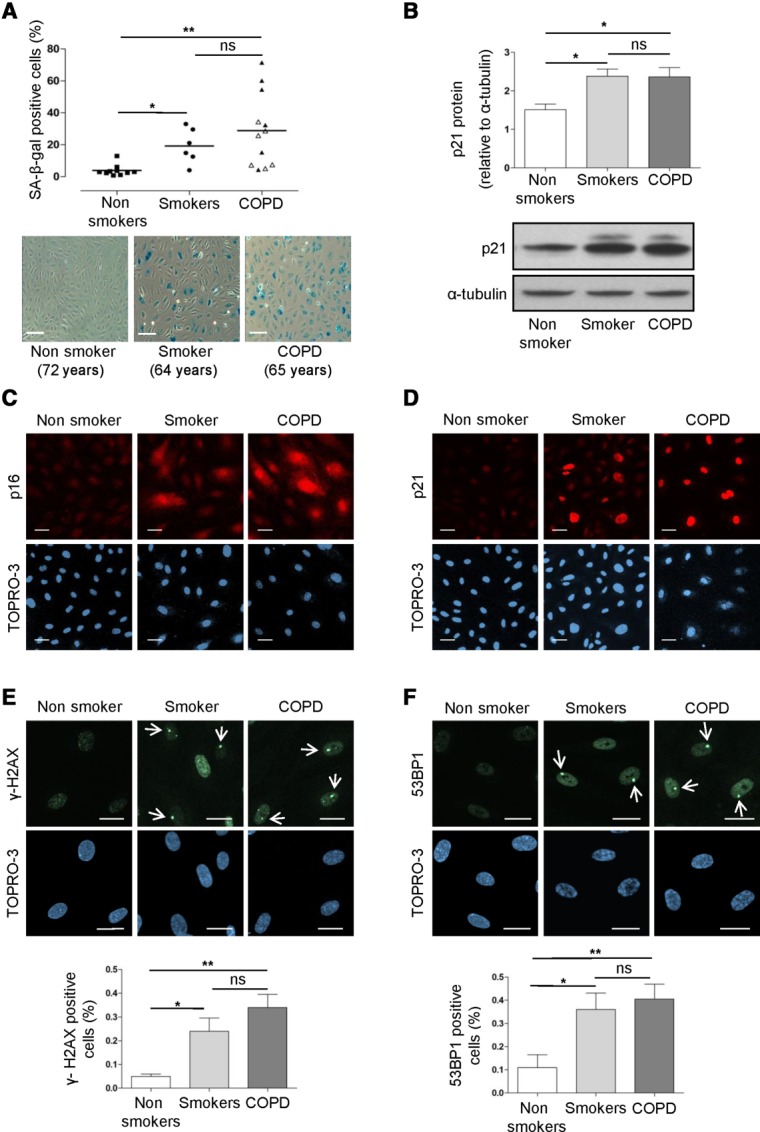
Increased senescence and DNA damage in smokers and COPD patients. SA-β-gal activity, p21, and p16 were assessed as markers of cellular senescence. (A): Quantification of SA-β-gal activity was assessed in blood outgrowth endothelial cells (BOEC) from healthy nonsmokers (*n* = 10), healthy smokers (*n* = 6), and COPD patients (*n* = 12), by counting the number of blue (senescent) cells relative to the total cell number and expressed as a percentage (%). BOEC from healthy smokers and COPD patients exhibited significantly increased senescence compared to healthy nonsmokers. Representative images from a healthy nonsmoker (72 years old), a healthy smoker (64 years old), and a COPD patient (65 years old) are shown (scale bars = 100 μm). (B): p21 protein levels were measured by Western blotting in whole cell lysates from BOEC isolated from healthy nonsmokers (*n* = 3), healthy smokers (*n* = 3), and COPD patients (*n* = 3). α-tubulin was used as loading control. p21 protein levels were significantly increased in smokers and COPD patients compared to nonsmokers. Representative images of Western blots are shown. (C, D): Immunofluorescence staining of BOEC for p16 (red, left panels) and p21 (red, right panels). TOPRO-3 (blue) was used as nuclear staining. Representative images from the three groups are shown. (E, F): DNA damage was assessed by measuring two markers of DSB formation, the γ-H2AX (left panels) and 53BP1 (right panels) by immunofluorescence staining (*n* = 3–5). Representative images from the three groups are shown. The number of cells with distinct nuclear immunofluorescent foci (see arrows) relative to the total cell number was counted in two to four optic fields, using a ×20 and ×40 objective lens (scale bars = 20 μm). An increased number of cells with focal nuclear staining of γ-H2AX was observed in healthy smokers and COPD patients compared to healthy nonsmokers (E, bottom). An increased number of cells with focal nuclear staining of 53BP1 was observed in healthy smokers and COPD patients compared to healthy nonsmokers (F, bottom). *, *p* < .05; **, *p* < .01 (open triangle: ex-smokers, black triangle: current smokers). Abbreviations: COPD, chronic obstructive pulmonary disease; SA-β-gal, senescence-associated-β-galactosidase; 53BP1, 53 binding protein 1.

### BOEC from Healthy Smokers and COPD Patients Show Increased Senescence

Increased cellular senescence has been described in lung biopsies from COPD patients and in human atherosclerotic plaques. BOEC from nonsmokers, healthy smokers, and COPD patients were stained for SA-β-gal. BOEC from healthy smokers and COPD patients displayed significantly increased SA-β-gal activity compared to healthy nonsmokers (Fig. 2A). We also measured p21 and p16 as alternative markers of senescence. Expression of both p21 and p16 was increased in BOEC from healthy smokers and COPD patients compared to healthy nonsmokers (Fig. 2B–2D), confirming the increased senescence in BOEC from healthy smokers and COPD patients. There was no correlation between SA-β-gal activity or p21 expression and age in all samples (Spearman *r*: .352, *p* = .06 and Spearman *r*: −.133, *p* = .7, respectively), indicating that the increased senescence observed in BOEC from healthy smokers and COPD patients is age-independent.

### DDR Is Increased in BOEC from Healthy Smokers and COPD Patients

Activation of the ATM-dependent DNA damage signaling pathway contributes to oxidative stress-induced premature senescence in endothelial cells 60. Mediators of DNA repair via ATM activation are phosphorylated histone H2AX at serine 139 (γ-H2AX) 61 and 53BP1 62; both mark the DSB in DNA and appear as distinct nuclear foci by immunofluorescent microscopy 36,61,62. The presence of these foci can be used to assess DNA damage and ATM activation, as shown in HUVEC treated with H_2_O_2_, which induces oxidative-stress premature senescence 60 (Supporting Information Fig. 3A, 3B).

**Figure 3:**
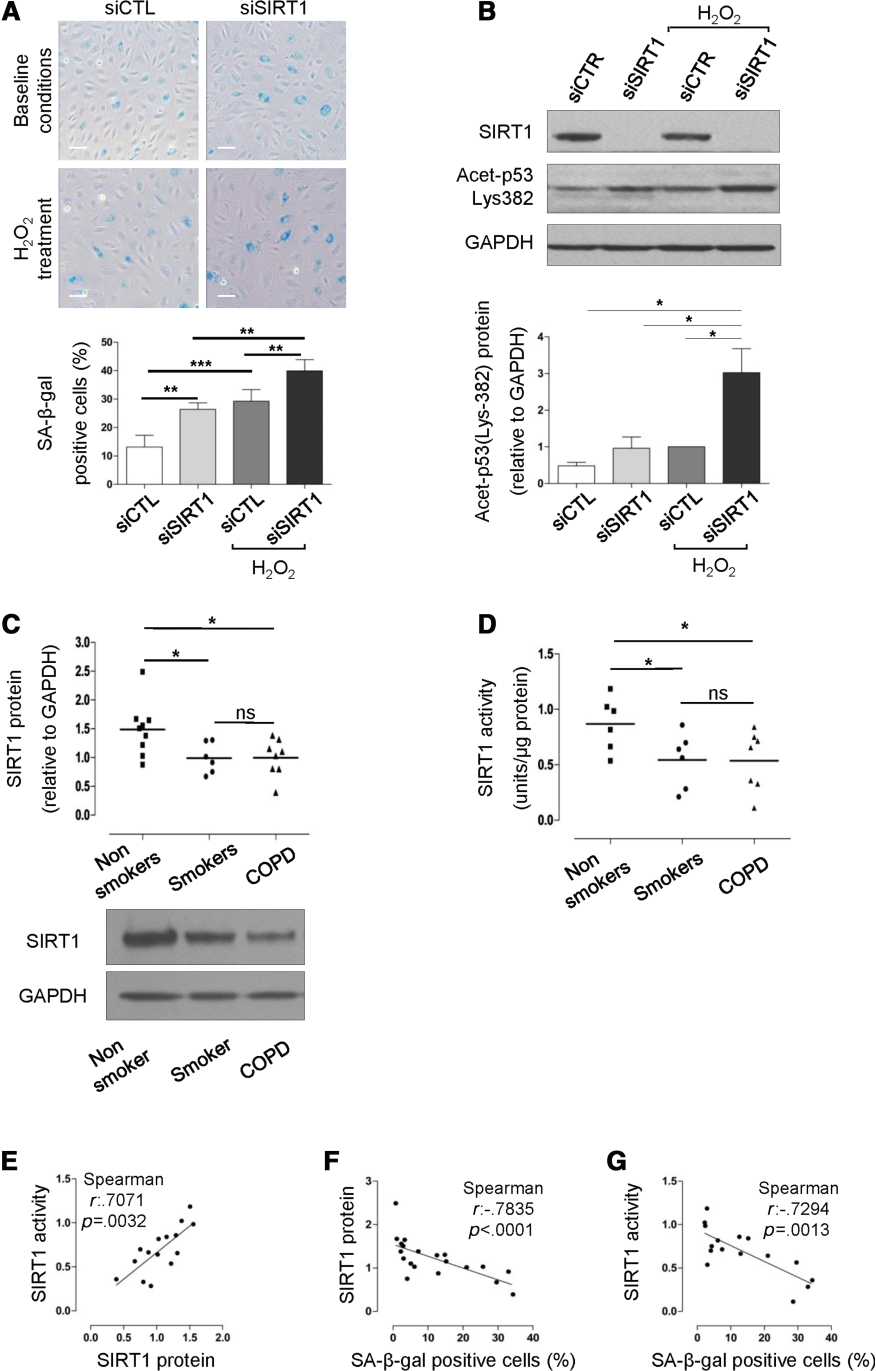
SIRT1 regulates senescence in blood outgrowth endothelial cells (BOEC); Decreased SIRT1 levels and activity in BOEC from healthy smokers and COPD patients compared to healthy nonsmokers. (A, B): BOEC from healthy nonsmokers (*n* = 4) were transfected with small interfering RNA (siRNA) against SIRT1 or control siRNA for 48 hours. Cells were exposed to 50 μmol/L H_2_O_2_ for 1 hour and cultured for 48 additional hours to induce oxidative-stress premature senescence. Cellular senescence was assessed by SA-β-gal activity. Protein levels of SIRT1 and acetylation of p53 at Lys-382 were measured by Western blotting. GAPDH was used as loading control. Inhibition of SIRT1 in BOEC induced increased SA-β-gal activity and acetylation of p53 at Lys-382, both under baseline and oxidant conditions. (C): SIRT1 protein levels were measured in BOEC from healthy nonsmokers (*n* = 9), healthy smokers (*n* = 6), and patients with COPD (*n* = 8) by Western blotting (representative image shown). GAPDH was used as loading control. SIRT1 protein levels were significantly reduced in smokers and COPD patients compared to nonsmokers. (D): SIRT1 activity was measured in nuclear extracts from BOEC isolated from healthy nonsmokers (*n* = 6), healthy smokers (*n* = 6), and COPD patients (*n* = 7) using a SIRT1 fluorescent activity assay kit. SIRT1 activity was significantly reduced in healthy smokers and COPD patients compared to healthy nonsmokers. (E–G): SIRT1 protein levels correlated with SIRT1 activity in samples from all groups. SIRT1 protein levels and activity negatively correlated with SA-β-gal activity in samples from all groups (scale bars = 100 μm) *, *p* < .05; **, *p* < .01; ***, *p* < .001. Abbreviations: COPD, chronic obstructive pulmonary disease; SA-β-gal senescence-associated-β-galactosidase; SIRT1, sirtuin-1.

An increased number of BOEC with distinct γ-H2AX and 53BP1 nuclear foci were observed in smokers and COPD patients compared to healthy nonsmokers (Fig. 2E, 2F). In particular, 53BP1 foci were large and resembled the “53BP1 nuclear bodies” which have been shown to accumulate at chromosomal fragile sites to protect them against erosion 62. There was no correlation between γ-H2AX or 53BP1 and age in all samples (Spearman *r*: .334, *p* = .345 and Spearman *r*: .333, *p* = .318, respectively), indicating that the increased DNA damage in BOEC from healthy smokers and COPD patients is age-independent. Both γ-H2AX and 53BP1 expression strongly correlated with SA-β-gal activity (Spearman *r*: 1.00, *p* < .0001 and Spearman *r*: .95, *p* < .0001 respectively), as well as with smoking pack-years (Table1) in all groups (Spearman *r*: .9633, *p* < .0001 and Spearman *r*: .8808, *p* = .0003, respectively), suggesting a causative link between DNA damage and senescence, possibly due to cigarette-smoke exposure.

### SIRT1 Regulates Senescence in BOEC via Deacetylation of p53; SIRT1 Protein Levels and Activity Are Reduced In Healthy Smokers and COPD Patients

Numerous studies describe the protective role of SIRT1 against endothelial senescence 53,63–65, which we confirmed in HUVEC by inhibiting SIRT1 expression with RNA interference. Treatment with siRNA for SIRT1 and control siRNA was performed in baseline conditions as well as in conditions of oxidative stress to induce premature senescence. As expected, abrogation of SIRT1expression in HUVEC resulted in increased senescence in both conditions (Supporting Information Fig. 4). We next confirmed the protective role of SIRT1 against senescence in BOEC, as inhibition of SIRT1 increased SA-β-gal activity in SIRT1-deficient BOEC compared to control siRNA-treated cells, both in baseline and oxidant conditions (Fig. 3A).

**Figure 4:**
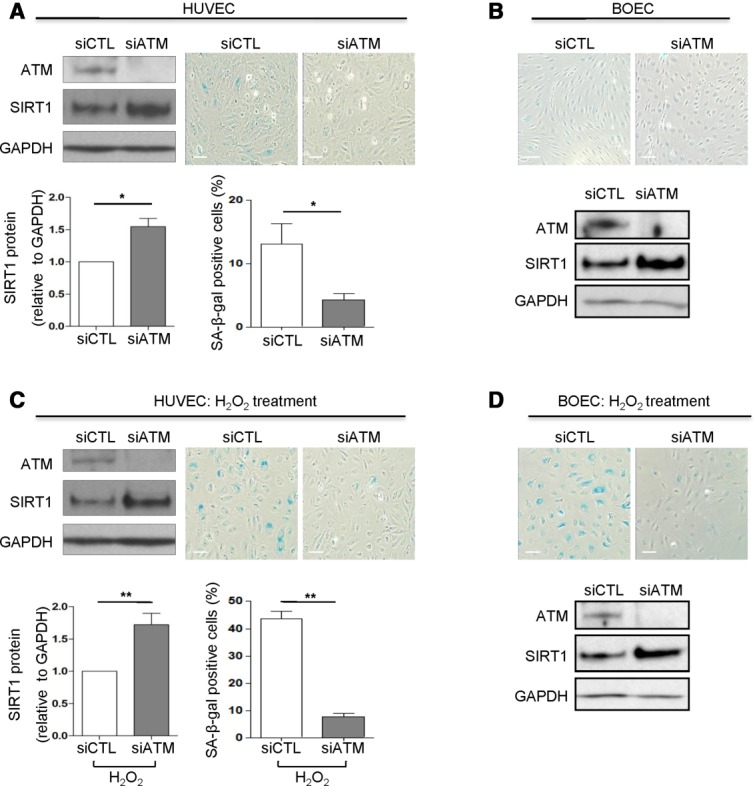
Inhibition of ATM signaling upregulates SIRT1 levels and suppresses endothelial senescence induced by oxidative stress. (A): HUVEC were transfected with small interfering RNA (siRNA) against ATM or control siRNA and grown in low serum medium for 72 hours. ATM and SIRT1 protein levels were measured by Western blotting. GAPDH was used as loading control. Cellular senescence was assessed by SA-β-gal activity. SIRT1 protein levels were significantly increased (bottom left) and senescence was significantly reduced (bottom right) in ATM-deficient cells compared to controls (*n* = 4–5). Representative images of Western blots and SA-β-gal activity are shown. (B): BOEC from healthy nonsmokers were treated and assessed as described in panel (A). (C): After 48 hours of siRNA treatment for ATM and control, HUVEC were exposed to 50 μmol/L H_2_O_2_ for 1 hour and cultured for 48 additional hours to induce oxidative-stress premature senescence. Inhibition of ATM in HUVEC by siRNA induced increased SIRT1 protein levels (*n* = 3–6). ATM silencing also caused a reduction in SA-β-gal activity in cells exposed to conditions of oxidative stress. Representative images of Western blots and SA-β-gal staining are shown. (D): BOEC from healthy nonsmokers were treated and assessed as described in panel (C) to induce oxidative-stress premature senescence (scale bars = 100 μm) *, *p* < .05; **, *p* < .01. Abbreviations: ATM, ataxia telangiectasia mutated; BOEC, blood outgrowth endothelial cells; HUVEC, human umbilical vein endothelial cells; SA-β-gal senescence-associated-β-galactosidase; SIRT1, sirtuin-1.

SIRT1 protein levels were significantly reduced in BOEC from healthy smokers and COPD patients compared to healthy nonsmokers (Fig. 3C). Similarly, SIRT1 activity was significantly reduced in cells from healthy smokers and COPD patients compared to healthy nonsmokers (Fig. 3D), with a significant correlation between SIRT1 protein levels and activity (Fig. 3E). No correlation between SIRT1 protein levels or activity and age of the subjects was found (Spearman *r*: .01089, *p* = .96 and Spearman *r*: −.2038, *p* = .42, respectively). There was a significant negative correlation between both SIRT1 protein levels and activity and SA-β-gal staining in all samples (Fig. 3F, 3G), suggesting that reduced SIRT1 expression was associated with the increased senescence in BOEC from healthy smokers and COPD patients.

SIRT1 has been shown to inhibit senescence by targeting p53, a transcription factor that becomes acetylated at Lys-382 as a result of DNA damage. This leads to enhanced p53 binding to DNA and transcription of genes that cause cell cycle arrest, senescence, or apoptosis 53,66. Inhibition of SIRT1 expression in BOEC, which caused increased senescence, also resulted in increased acetylation of p53 at Lys-382, suggesting that the protective effect of SIRT1 against senescence in BOEC may be in part mediated by deacetylation of p53 (Fig. 3B).

### ATM Regulates SIRT1 Protein Expression and Endothelial Senescence Induced by Oxidative Stress

SIRT1 is involved in ATM activation and downstream signaling pathways promoting cell survival and DNA repair 67,68 and in turn ATM regulates SIRT1 activity 69. To test whether ATM could also regulate SIRT1 levels, we inhibited ATM expression by siRNA in HUVEC. Inhibition of ATM caused a significant increase in SIRT1 mRNA and protein levels and a reduction in SA-β-gal activity compared to control cells (Supporting Information Fig. 5; Fig. 4A). Silencing of ATM also resulted in increased SIRT1 protein levels in BOEC (Fig. 4B); however, its effect on senescence was minimal because BOEC from healthy nonsmokers exhibited a low-grade of baseline senescence (Fig. 4B).

**Figure 5:**
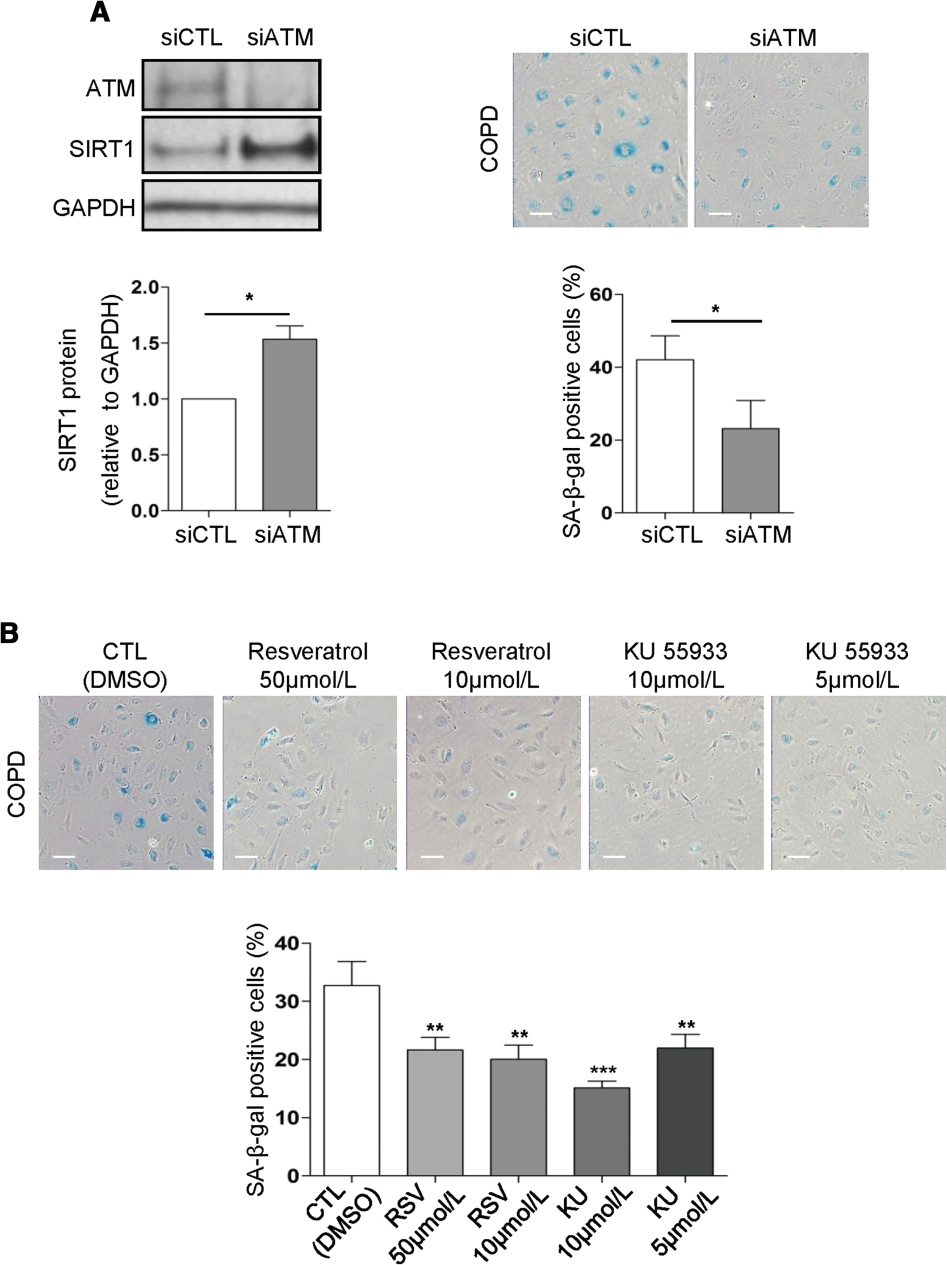
Inhibition of ATM suppresses senescence in blood outgrowth endothelial cells (BOEC) from COPD patients; treatment with SIRT1 activators and ATM inhibitors. (A): BOEC from COPD patients were transfected with siRNA and grown in low serum medium for 72 hours. SIRT1 and ATM were measured by Western blotting and cellular senescence by SA-β-gal activity. SIRT1 protein levels were significantly increased (bottom left), and senescence was significantly reduced (bottom right) in ATM-deficient BOEC (*n* = 3). Representative images of Western blots and SA-β-gal staining are shown. (B): BOEC from COPD patients were seeded in basal medium (plus 10% fetal bovine serum) for 24 hours. BOEC were treated with different concentrations of the SIRT1 activator RSV (10 and 50 μmol/L) or the selective ATM inhibitor KU (5 and 10 μmol/L) for 48 hours. Treatment with both concentrations of RSV or KU significantly reduced the grade of senescence compared to control treated BOEC from COPD patients (*n* = 4) (scale bars = 100 μm) *, *p* < .05; **, *p* < .01; ***, *p* < .001. Abbreviations: ATM, ataxia telangiectasia mutated; COPD, chronic obstructive pulmonary disease; DMSO, Dimethyl sulfoxide; KU, KU-55933; RSV, resveratrol; SA-β-gal senescence-associated-β-galactosidase; SIRT1, sirtuin-1.

We next examined whether regulation of SIRT1 and senescence by ATM also applies under conditions of oxidative stress, which induces DNA damage and premature endothelial senescence and reduces SIRT1 protein levels 53 (Supporting Information Fig. 3A–3C). Inhibition of ATM, in H_2_O_2_-treated HUVEC induced a significant increase in SIRT1 protein levels and reduction in senescence compared to control siRNA-treated cells (Fig. 4C). The same mechanism was confirmed in BOEC from healthy nonsmokers, where abrogation of ATM expression by siRNA increased SIRT1 protein levels and inhibited the oxidative stress-induced senescence (Fig. 4D).

We next examined whether the same occurs in BOEC from COPD patients, which display increased senescence in baseline conditions. Interestingly, inhibition of ATM expression in BOEC from COPD patients upregulated SIRT1 levels and reduced senescence (Fig. 5A), suggesting that SIRT1 upregulation through ATM inhibition could be a therapeutic target in these patients.

### SIRT1 Activators and ATM Inhibitors Reduce Senescence in BOEC

SIRT1 activators like resveratrol and selective ATM inhibitors like KU-55933 have been shown to reduce stress-induced premature senescence in HUVEC 60,63. Therefore, we tested whether pharmacological treatment with SIRT1 activators and ATM inhibitors could inhibit senescence in BOEC from COPD patients. BOEC isolated from COPD patients were treated with two different concentrations of resveratrol (Sigma-Aldrich Company Ltd.) or KU-55933 (Calbiochem, Merck Chemicals Ltd., Nottingham, U.K., http://www.merck.com). Treatment with both doses of resveratrol and KU-55933 for 48 hours significantly inhibited the increased senescence observed in BOEC from COPD patients (Fig. 5B). In most cases, higher doses of both resveratrol (50 μmol/L) and KU-55933 (10 μmol/L) reduced the confluence of the cells, indicating possible toxicity of these drugs at higher doses. We confirmed that the protective effect of resveratrol against senescence is mediated by SIRT1 activation, as inhibition of oxidative stress-induced premature senescence in SIRT1-deficient HUVEC and BOEC was abolished by treatment with resveratrol (Supporting Information Fig. 6). Our results indicate that SIRT1 activators and possibly selective ATM inhibitors could be used therapeutically to inhibit the increased senescence in circulating endothelial progenitors in COPD patients with possible beneficial effects on endothelial function and CVD.

**Figure 6:**
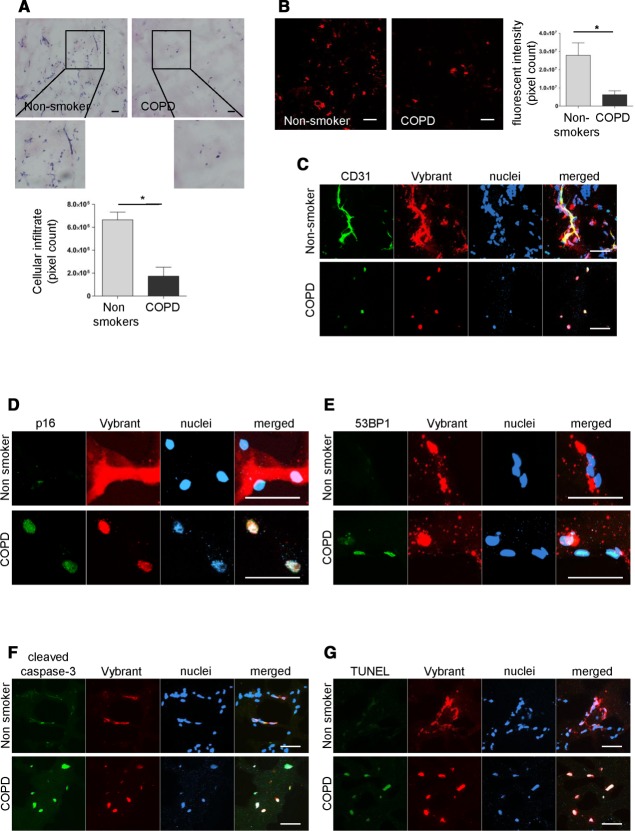
Senescent blood outgrowth endothelial cells (BOEC) from COPD patients show reduced angiogenic ability and increased apoptosis. (A–G): BOEC from nonsmokers (*n* = 4) or COPD patients (*n* = 3) were labeled with Dil-Vybrant cell solution and were subcutaneously injected in severe combined immunodeficiency mice. After 7 days, plugs were harvested, frozen, cryosectioned, and stained with hematoxylin and eosin (H&E) (A) or analyzed by immunofluorescence microscopy (B, C). Experiments were performed for each sample in duplicate. Cellular infiltrate was quantified in H&E sections (A, bottom) and Vybrant positive cells were quantified from Z-stack images taken with ×20 objective lens (B, right). (C): BOEC from nonsmokers formed capillary-like structures in the Matrigel plugs. Immunofluorescent analysis for CD31 staining (green) revealed lining of the Vybrant positive cells and formation of capillary-like structures in the Matrigel plugs with BOEC from nonsmokers but not in COPD samples. (D–G): Immunofluorescent analysis for p16 staining (green, top left), 53BP1 (green, top right), cleaved caspase-3 (green, bottom left), and TUNEL staining (green, bottom right) showed increased expression of all markers in BOEC from COPD patients compared to nonsmokers (scale bars = 50 μm). Abbreviations: COPD, chronic obstructive pulmonary disease; 53BP1, 53 binding protein 1.

### BOEC from COPD Patients Show Reduced Angiogenic Ability, and Increased DNA Damage, Senescence and Apoptosis

BOEC exhibit *in vivo* angiogenic ability and form functional vessels when in appropriate matrix and implanted into immunocompromised mice (mice with severe combined immunodeficiency [SCID]) 3,70,71. To test whether the *in vitro* BOEC dysfunction was reflected *in vivo*, we studied the *in vivo* angiogenic capacity of these cells after injection into SCID mice. BOEC from nonsmokers and COPD patients were labeled with the Vybrant DiI Cell-Labeling Solution, mixed with Matrigel, and subcutaneously injected in SCID mice, as previously described 72. Interestingly, Matrigel plugs with BOEC from COPD patients showed decreased cellular content and capillary-like structures compared to healthy nonsmokers (Fig. 6A). An increased number of Vybrant positive cells was found in the Matrigel plugs with BOEC from nonsmokers compared to those with BOEC from COPD subjects (Fig. 6B). Immunofluorescent analysis revealed lining and colocalization of CD31 and Vybrant positive cells and formation of capillary-like structures in the Matrigel plugs with BOEC from nonsmokers but not in COPD samples (Fig. 6C).

Increased DDR and senescence (measured by expression of 53BP1 and p16) was found in Vybrant positive cells in the Matrigel plugs with BOEC from COPD patients compared to nonsmokers (Fig. 6D, 6E), in line with the *in vitro* results. Endothelial senescence is also linked to increased apoptosis 73,74 and oxidant conditions have been shown to induce increased apoptosis and reduced tube-forming ability in BOEC 75. We therefore investigated the presence of apoptotic cells by staining for cleaved caspase-3 and TUNEL, and found increased BOEC apoptosis in the COPD group compared to nonsmokers (Fig. 6F, 6G). Therefore, the *in vivo* data demonstrate that BOEC from COPD patients display impaired angiogenic ability compared to nonsmokers, linked to increased DNA damage, senescence, and apoptosis.

## DISCUSSION

In this study, we demonstrate increased DNA damage and senescence in progenitor cells of the endothelial lineage in smokers and COPD patients. This study provides strong evidence of epigenetic alterations linked to cigarette smoke-oxidative stress, demonstrating reduced SIRT1 expression via the ATM mediated DDR pathway, and providing new insights into the molecular mechanisms involved in endothelial dysfunction in smokers and COPD patients.

Increased DNA damage and senescence have previously been shown in the peripheral lung of normal smokers and in a greater degree in COPD patients compared to nonsmoking controls 34,37,76. This likely reflects the lung injury induced by cigarette smoke-oxidative stress 38,39,77,78. Other studies show evidence of increased senescence and telomere shortening in circulating white blood cells in smokers and COPD patients 79–81, providing evidence for widespread systemic cellular senescence and confirming the proaging effects of smoking. In our study, we show for the first time increased DNA damage and senescence in BOEC from smokers and COPD patients compared to healthy nonsmokers, which were independent of the subjects' age. Therefore, our findings suggest accelerated aging of BOEC in healthy smokers and COPD patients and possibly reflect a systemic effect of cigarette smoke-oxidative stress on circulating endothelial progenitors. Interestingly, BOEC from smokers and COPD patients, expanded in culture in nonoxidant conditions, retained a senescent and dysfunctional phenotype. Among the possible mechanisms responsible for this may be transmission of the DNA damage to the daughter cells and/or epigenetic alterations linked to increased senescence.

BOEC have been shown to contribute to endothelial homeostasis and repair in areas of vascular damage, as they possess a robust proliferative potential and can incorporate into and form new blood vessels 82. Several studies indicate that DNA damage and endothelial senescence contribute to vascular dysfunction and the development of atherosclerosis 16,17,43,83,84. BOEC have been shown to be sensitive to oxidative stress, and when treated with oxidants they show reduced clonogenic capacity, increased apoptosis, and reduced vessel formation *in vivo* 75. We demonstrated that BOEC from COPD patients show reduced ability to form a capillary network in an *in vivo* angiogenesis assay, which was also confirmed in BOEC from a smoker volunteer (Supporting Information Fig. 7). Based on the results of our study, we propose that circulating BOEC from smokers and COPD patients, which show increased DNA damage and senescence, are ineffective in repairing vascular damage and maintaining vascular integrity and may be involved in endothelial dysfunction in these groups, leading to an increased severity of CVD.

The ATM mediated DDR pathway regulates cellular senescence 30,31,60. We showed that inhibition of the ATM pathway results in upregulation of SIRT1 expression. The protective role of SIRT1 overexpression against endothelial senescence via deacetylation of p53 has been clearly demonstrated in previous studies 53 and we also confirmed it in BOEC. SIRT1 levels and activity are significantly decreased in peripheral lung tissue and macrophages from COPD patients, indicating a possible involvement of SIRT1 in the pathogenesis of COPD 50,51. In this study, we observed significantly lower levels of SIRT1 protein and activity in BOEC from healthy smokers and COPD patients compared to healthy nonsmokers. In all subjects, the levels of SIRT1 protein and activity negatively correlated with the grade of senescence, suggesting that the increased BOEC senescence in smokers and COPD patients could be linked to reduced SIRT1 expression, secondary to cigarette smoke-induced oxidative stress activation of ATM. Importantly, we showed that the senescent phenotype of BOEC from smokers and COPD patients can be reversed by the SIRT1 activator resveratrol or an ATM inhibitor. Since ATM and SIRT1 proteins are currently targets for drug development, with compounds already in clinical studies 85,86, our data provides further rationale for exploring the effects of these compounds in these patients.

Previous studies have found reduced numbers of circulating EPC in healthy smokers 26 and COPD patients 24,27; however, these findings were not confirmed by others 87,88. In our study, we did not find a difference in the number of endothelial progenitors, measured as BOEC colonies per milliliter blood, in healthy smokers or COPD group. The discrepancy between studies may be due to the different cell populations examined. Previous studies have assessed the number of circulating EPC by measuring the endothelial cell-colony forming units (EC-CFU) in mononuclear cell cultures, or the number of mononuclear cells positive for CD34/CD133/VEGFR-2 surface markers by flow cytometry. However, EC-CFU have been shown to represent a heterogeneous population including myeloid cells which can express endothelial characteristics in culture 89, while the CD34/CD133/VEGFR2 positive subset of mononuclear cells was found to be enriched with hematopoietic colony-forming cells that cannot differentiate into mature endothelial cells *in vitro* or *in vivo* 90. Currently, there is no unique marker to identify circulating EPC and, as Yoder and Ingram have suggested 82, it might be helpful to avoid the term EPC and refer instead to the specific cell population under investigation.

In our study, the majority of our COPD patients had moderate disease (GOLD2). We did not recruit COPD patients with very severe disease (GOLD4), hypoxemia, and other major comorbidities, which could possibly have a further impact on endothelium and on BOEC function. We also excluded COPD patients and smokers with overt CVD, as these subjects would be more likely to show abnormalities in BOEC function. Even though it is important to see the effect of all these factors on the endothelium, our aim in this study was to identify the dysfunction imposed by cigarette smoke on the endothelium with the least confounding factors, such as hypoxia and the effect of medications. Future studies will expand our work to involve COPD patients with more severe disease and other co-morbidities, such as smokers with CVD.

## Conclusion

In summary, we have provided evidence of increased DNA damage and endothelial senescence in smokers and COPD patients, processes that are known to increase the risk of atherosclerosis and CVD. We have also identified a molecular pathway whereby DNA damage and activation of ATM regulates SIRT1 expression, suggesting a mechanism for DNA damage-mediated endothelial senescence and dysfunction. The use of circulating BOEC allows the investigation of underlying molecular mechanisms affecting the endothelium. The effect of cigarette smoke exposure on endothelial progenitors, which are thought to have regenerative properties for the vascular system, provides a better understanding of the CVD seen in smokers and patients with COPD.

## Supporting Information

Supporting Information Figure 1. BOEC isolation. (A-B) Colonies of BOEC appeared with a characteristic cobblestone morphology between 7-22 days and grew in culture. Representative images of a BOEC colony in culture over time. (C) Endothelial cells derived from the colonies were passaged for 2-3 weeks after appearance and grown to confluence, giving rise to morphologically homogeneous cultures which were expanded and used between P3 and P6. (D) Colonies appeared later in culture in healthy smokers compared to healthy non-smokers. (E) Number of BOEC colonies in healthy non-smokers, healthy smokers and patients with COPD. Peripheral blood mononuclear cells (PBMC) from healthy non-smokers (n=18), smokers with normal lung function (n=11) and COPD patients (n=20) were seeded in complete endothelial growth medium (EGM)-2 supplemented with 10% FBS, on type I collagen coated 6-well plates. In 2 non-smokers, 5 smokers and 6 COPD patients the assay for BOEC isolation was carried out twice since the first isolation procedure was unsuccessful. The number of discrete colonies appeared in PBMC culture between 7 and 22 days was counted relative to the number of the millilitres of blood received. There was no difference in the number of BOEC colonies per mL of blood between the three groups. (F-G) There was no difference in the number of the days that colonies grew in the culture before the first passage between the three groups, both when compared from the day of isolation, and from the day of the first colony appearance in culture. *p<0.05 (open triangle: ex-smokers, black triangle: current smokers).

Supporting Information Figure 2. BOEC characterisation. (A-C) BOEC were stained for VEGFR-2, VE-cadherin, VWF, PECAM, CD3, CD45. TOPRO-3 was used as nuclear stain (purple). BOEC were positive for the endothelial markers VEGFR-2, VE-cadherin, VWF and PECAM and negative for the lymphocytic CD3 and leukocytic CD45 markers.

Supporting Information Figure 3. Increased DNA damage and decreased SIRT1 levels in senescent endothelial cells due to oxidative stress. Human umbilical vein endothelial cells (HUVEC) were treated with 25μmol/L or 50 μmol/L H2O2 for 1hour and cultured for two additional days to induce oxidative stress-premature senescence. (A) Oxidative-premature senescence was confirmed by SA-β-gal staining. Quantification of SA-β-gal activity was assessed by counting the number of blue (senescent) cells relative to the total cell number. Results were expressed as a percentage. HUVEC (n=3) treated with 25μmol or 50μmol/L H2O2 exhibited increased senescence compared to untreated cells (scale bars 100μm). (B) DNA damage was assessed by measuring two markers of double-strand break (DSB) formation, the γ-H2AX and 53BP1, by immunostaining. HUVEC non-treated showed a homogeneous nuclear staining of γ-H2AX and 53BP1. HUVEC treated with 50μmol/L H2O2 showed increased focal nuclear staining of γ-H2AX and 53BP1 (scale bars 20μm). (C) SIRT1 protein levels were measured by Western blotting. GAPDH was used as loading control. HUVEC (n=2) treated with H2O2 exhibited decreased levels of SIRT1 compared to untreated cells. *p<0.05, **p<0.01.

Supporting Information Figure 4. Inhibition of SIRT1 expression induces endothelial senescence. Human umbilical vein endothelial cells (HUVEC) (n=3) were transfected with siRNA against SIRT1 or control siRNA for 48h. Cells were exposed to H2O2 (50μmol/L for 1 hour) and cultured for 48 additional hours (medium with no growth factors and 10% FBS) to induce oxidative-stress premature senescence. Cellular senescence was assessed by SA-β-gal activity. HUVEC treated with siRNA against SIRT1 showed increased senescence compared to control siRNA treated cells both in baseline and conditions of oxidative stress (scale bars 100μm). **p<0.01.

Supporting Information Figure 5. Inhibition of ATM signaling up-regulates SIRT1 mRNA levels. (A) Human umbilical vein endothelial cells (HUVEC) (n=3) were transfected with siRNA against ATM or control siRNA in low serum medium for 48h. SIRT1 mRNA levels were measured by RT-PCR. GAPDH was used as loading control. SIRT1 mRNA levels were significantly increased in ATM knock-down cells 48h post siRNA treatment. *p<0.05.

Supporting Information Figure 6. Resveratrol inhibits premature senescence due to oxidative stress via SIRT1 activation. (A) Human umbilical vein endothelial cells (HUVEC) were transfected with siRNA against SIRT1 or control siRNA for 48h. After pretreatment with resveratrol (10μM) or control medium for one hour, cells were exposed to H2O2 (50μmol/L for 1 hour) to induce oxidative-stress premature senescence, then cultured for 48 additional hours with resveratrol or control media. Cellular senescence was assessed by SA-β-gal activity. Protein levels of SIRT1 and acetylation of p53 at Lys-382 were measured by Western blotting. GAPDH was used as loading control. Resveratrol treatment caused a reduction of SA-β-gal activity only in control siRNA treated cells. The effect was abolished in SIRT1-deficient HUVEC. Increased acetylation of p53 at Lys-382 was maintained in SIRT1-deficient cells compared to control cells after treatment with resveratrol. (B) Blood outgrowth endothelial cells (BOEC) from healthy non-smokers were treated as described for HUVEC. Resveratrol treatment inhibited senescence in control siRNA treated cells but not in SIRT1-deficient cells, which maintained increased acetylation of p53 (Lys-382). SA-β-gal activity is displayed as percentage of SA-β-gal positive cells relative to the total number of cells, at the right bottom corner of the images (scale bars 100μm).

Supporting Information Figure 7. BOEC from smokers and COPD patients show reduced angiogenic ability. BOEC from non-smokers (n=4), smokers (n=1) and COPD patients (n=3) were labeled with Dil-Vybrant cell solution, and were subcutaneously injected in SCID mice. Seven-day-old plugs were harvested, frozen, cryosectioned, and analysed by immunofluorescence confocal microscopy. Experiments were performed for each sample in duplicate. Immunofluorescent analysis for CD31 staining (green) revealed lining of the Vybrant positive cells and formation of capillary-like structures in the Matrigel plugs with BOEC from non-smokers but not in smokers and COPD samples (scale bars 50μm).
