# Supplementary figures and images for: Dysfunction of Endothelial Progenitor Cells from Smokers and Chronic Obstructive Pulmonary Disease Patients Due to Increased DNA Damage and Senescence
Source: Stem Cells. 2013 Dec 3;31(12):2813–26. doi: 10.1002/stem.1488 (PMC4377082; doi:10.1002/stem.1488)

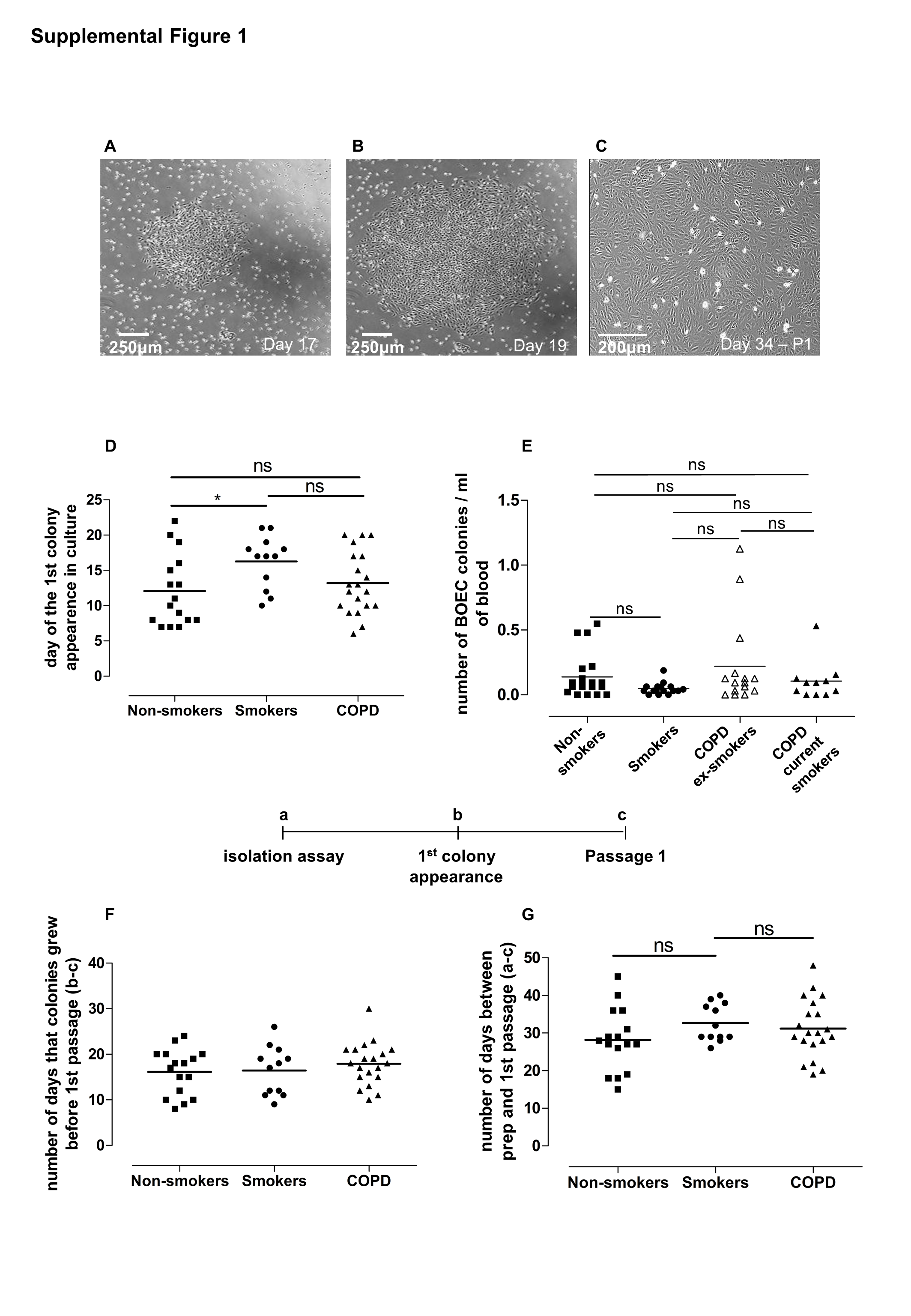

Supplement: Supplementary file 1 — Supporting Information Figure 1. BOEC isolation. (A-B) Colonies of BOEC appeared with a characteristic cobblestone morphology between 7-22 days and grew in culture. Representative images of a BOEC colony in culture over time. (C) Endothelial cells derived from the colonies were passaged for 2-3 weeks after appearance and grown to confluence, giving rise to morphologically homogeneous cultures which were expanded and used between P3 and P6. (D) Colonies appeared later in culture in healthy smokers compared to healthy non-smokers. (E) Number of BOEC colonies in healthy non-smokers, healthy smokers and patients with COPD. Peripheral blood mononuclear cells (PBMC) from healthy non-smokers (n=18), smokers with normal lung function (n=11) and COPD patients (n=20) were seeded in complete endothelial growth medium (EGM)-2 supplemented with 10% FBS, on type I collagen coated 6-well plates. In 2 non-smokers, 5 smokers and 6 COPD patients the assay for BOEC isolation was carried out twice since the first isolation procedure was unsuccessful. The number of discrete colonies appeared in PBMC culture between 7 and 22 days was counted relative to the number of the millilitres of blood received. There was no difference in the number of BOEC colonies per mL of blood between the three groups. (F-G) There was no difference in the number of the days that colonies grew in the culture before the first passage between the three groups, both when compared from the day of isolation, and from the day of the first colony appearance in culture. *p<0.05 (open triangle: ex-smokers, black triangle: current smokers). [file stem0031-2813-sd1.tif]

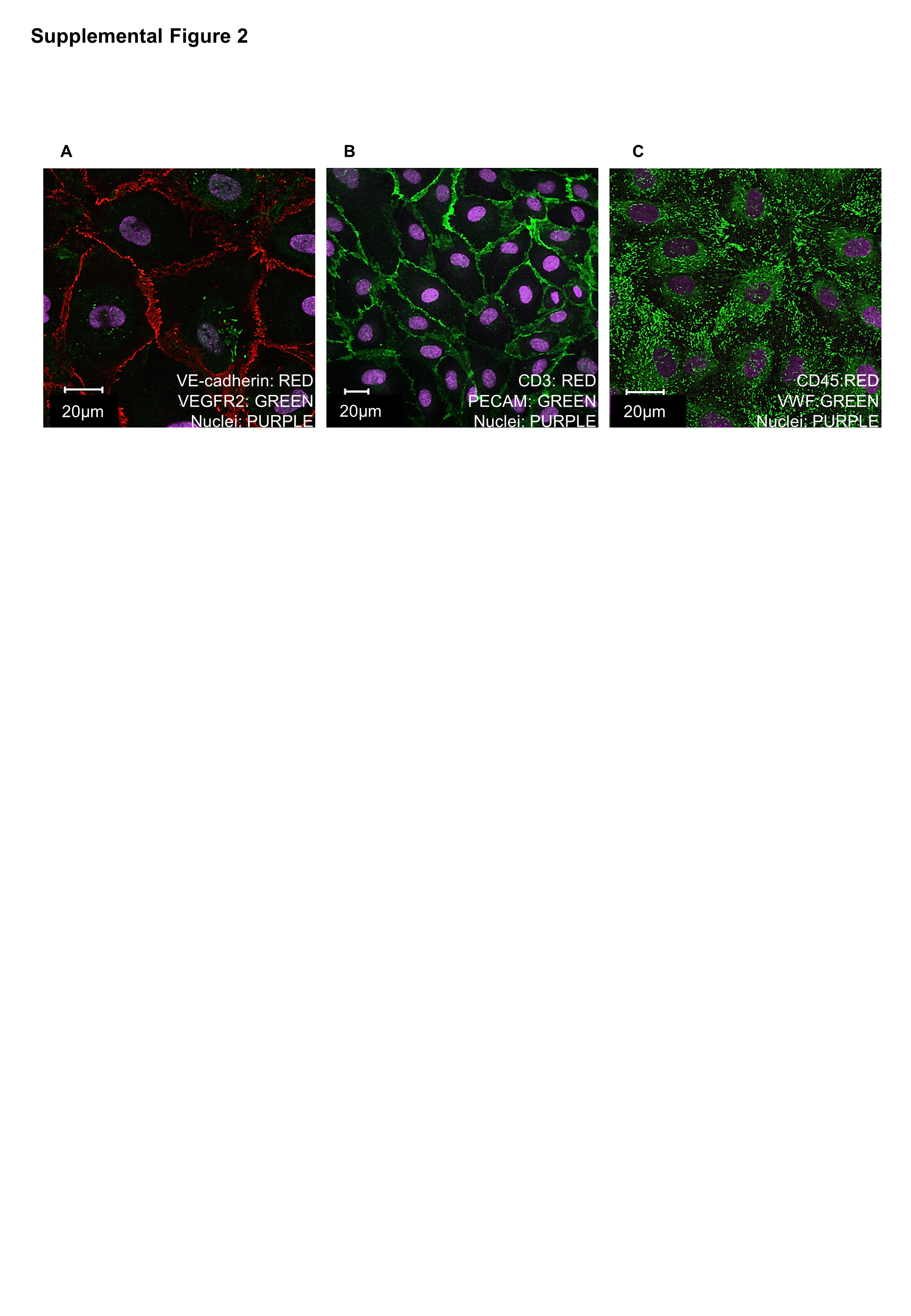

Supplement: Supplementary file 2 — Supporting Information Figure 2. BOEC characterisation. (A-C) BOEC were stained for VEGFR-2, VE-cadherin, VWF, PECAM, CD3, CD45. TOPRO-3 was used as nuclear stain (purple). BOEC were positive for the endothelial markers VEGFR-2, VE-cadherin, VWF and PECAM and negative for the lymphocytic CD3 and leukocytic CD45 markers. [file stem0031-2813-sd2.tif]

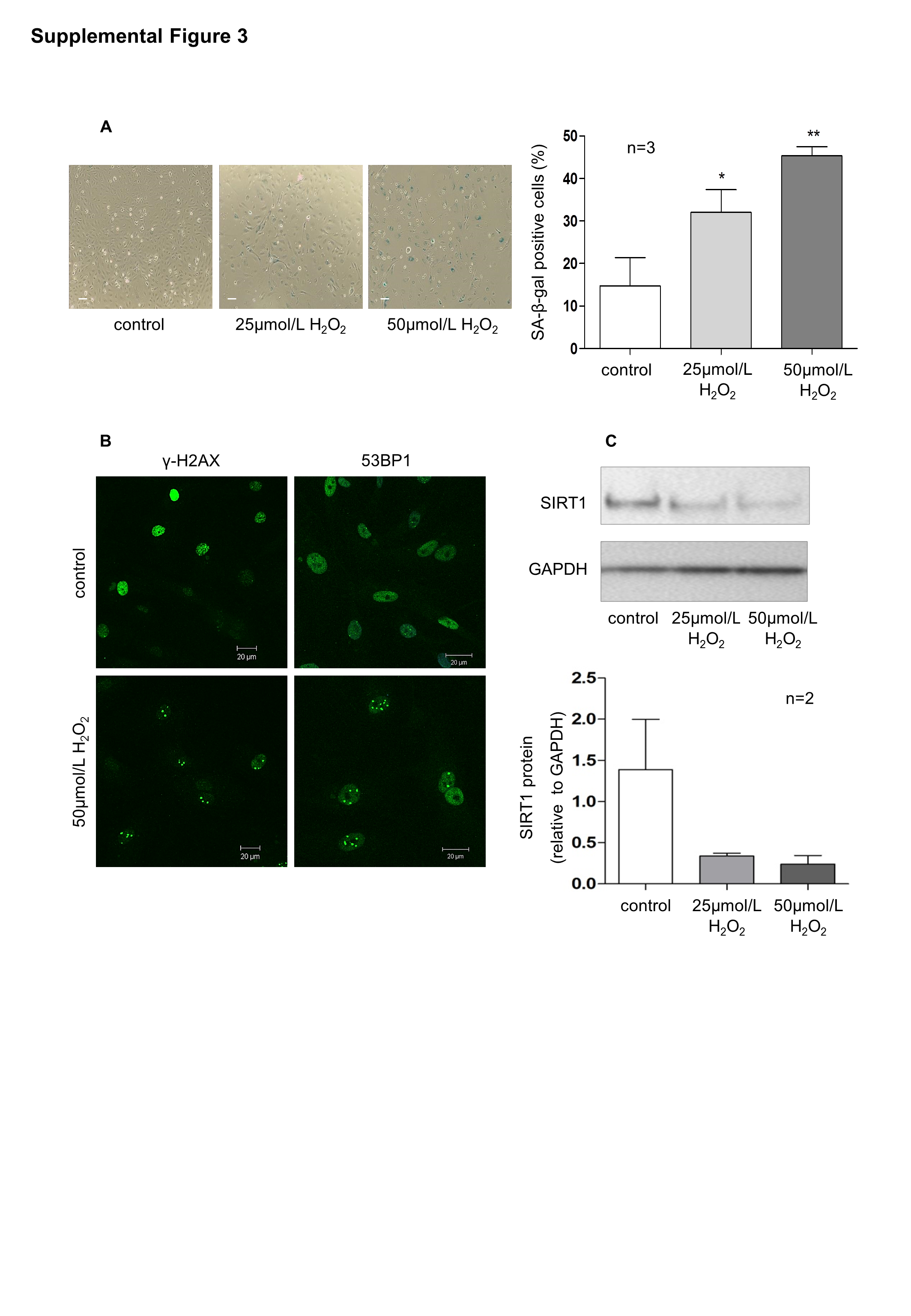

Supplement: Supplementary file 3 — Supporting Information Figure 3. Increased DNA damage and decreased SIRT1 levels in senescent endothelial cells due to oxidative stress. Human umbilical vein endothelial cells (HUVEC) were treated with 25μmol/L or 50 μmol/L H2O2 for 1hour and cultured for two additional days to induce oxidative stress-premature senescence. (A) Oxidative-premature senescence was confirmed by SA-β-gal staining. Quantification of SA-β-gal activity was assessed by counting the number of blue (senescent) cells relative to the total cell number. Results were expressed as a percentage. HUVEC (n=3) treated with 25μmol or 50μmol/L H2O2 exhibited increased senescence compared to untreated cells (scale bars 100μm). (B) DNA damage was assessed by measuring two markers of double-strand break (DSB) formation, the γ-H2AX and 53BP1, by immunostaining. HUVEC non-treated showed a homogeneous nuclear staining of γ-H2AX and 53BP1. HUVEC treated with 50μmol/L H2O2 showed increased focal nuclear staining of γ-H2AX and 53BP1 (scale bars 20μm). (C) SIRT1 protein levels were measured by Western blotting. GAPDH was used as loading control. HUVEC (n=2) treated with H2O2 exhibited decreased levels of SIRT1 compared to untreated cells. *p<0.05, **p<0.01. [file stem0031-2813-sd3.tif]

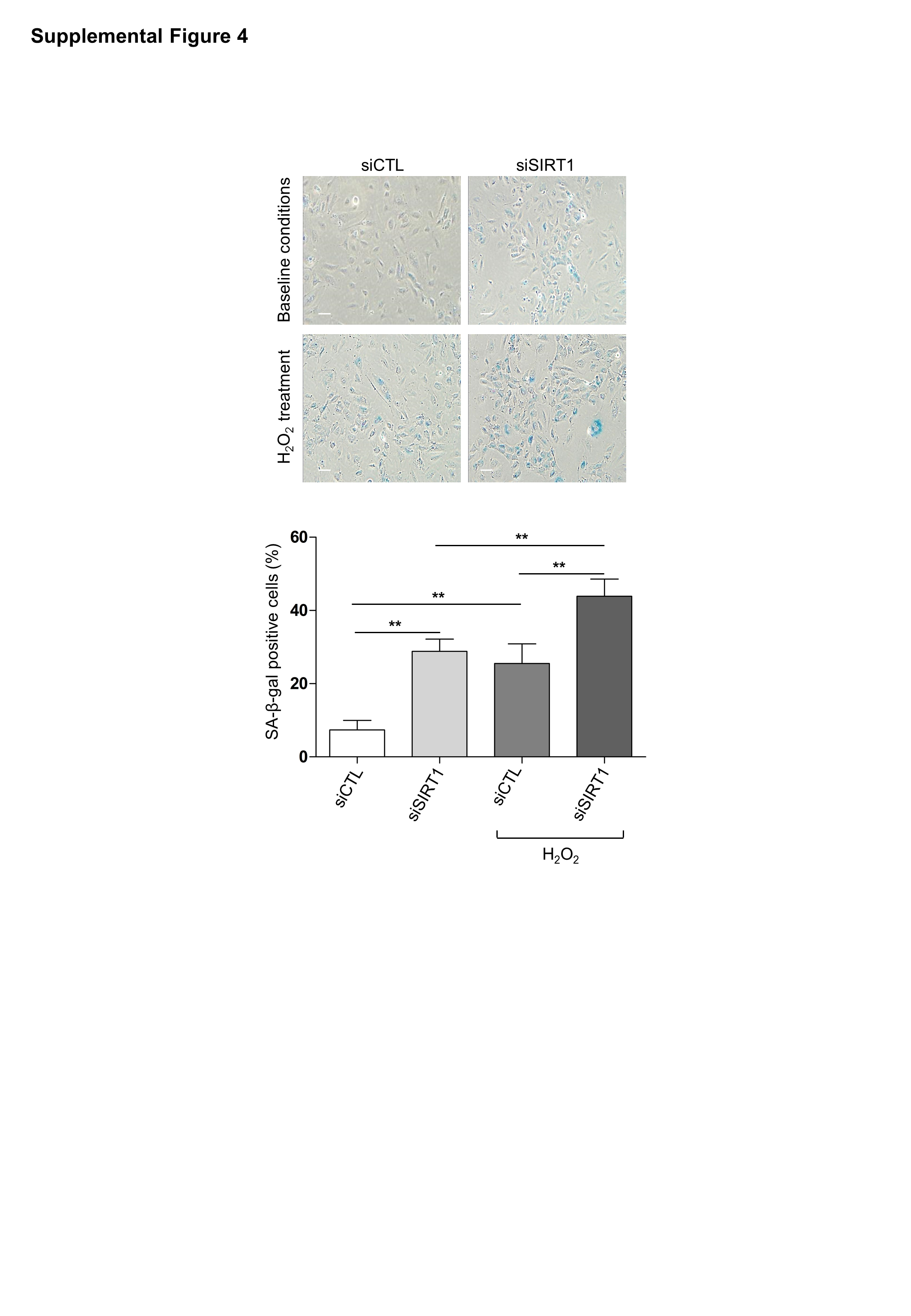

Supplement: Supplementary file 4 — Supporting Information Figure 4. Inhibition of SIRT1 expression induces endothelial senescence. Human umbilical vein endothelial cells (HUVEC) (n=3) were transfected with siRNA against SIRT1 or control siRNA for 48h. Cells were exposed to H2O2 (50μmol/L for 1 hour) and cultured for 48 additional hours (medium with no growth factors and 10% FBS) to induce oxidative-stress premature senescence. Cellular senescence was assessed by SA-β-gal activity. HUVEC treated with siRNA against SIRT1 showed increased senescence compared to control siRNA treated cells both in baseline and conditions of oxidative stress (scale bars 100μm). **p<0.01. [file stem0031-2813-sd4.tif]

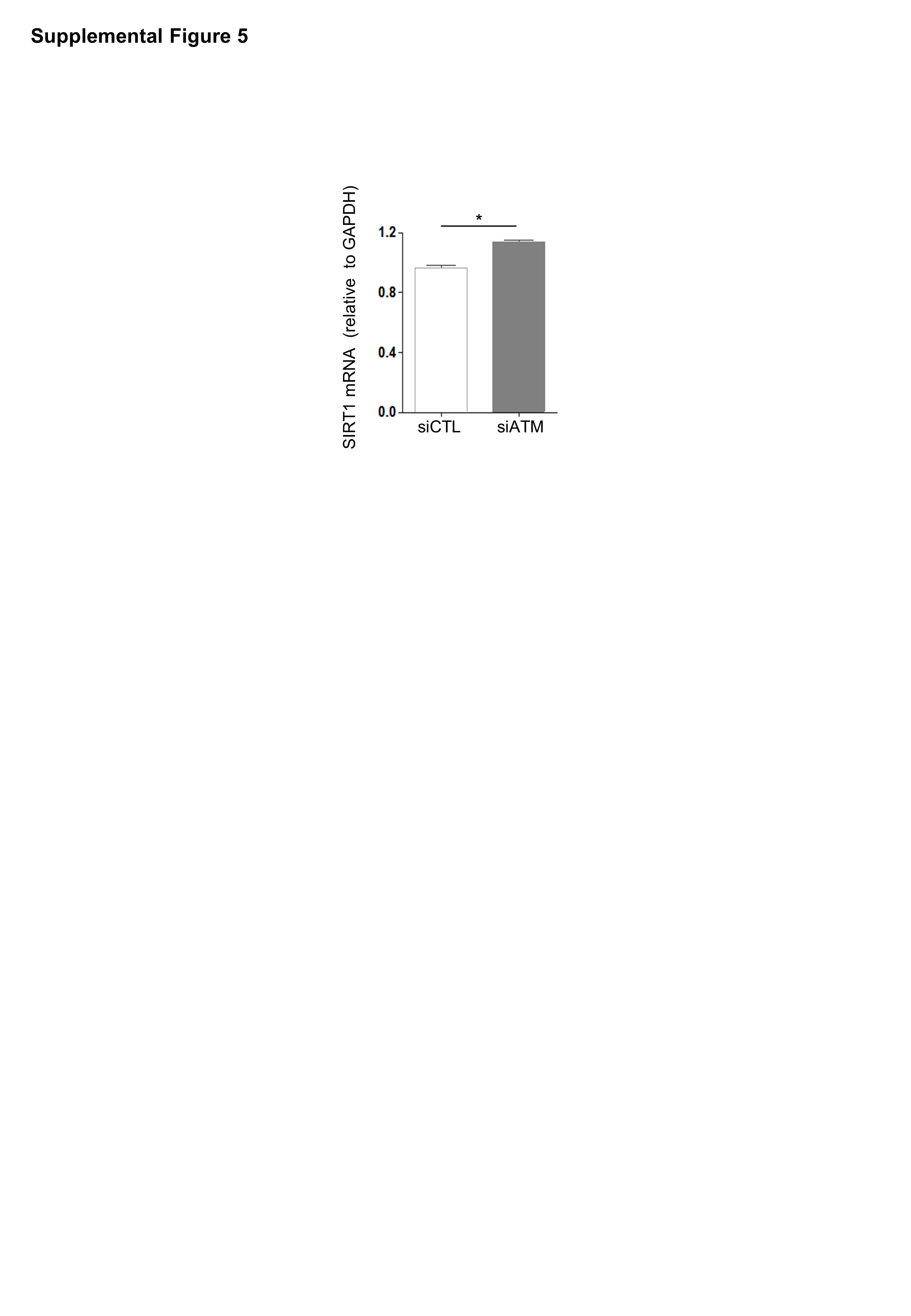

Supplement: Supplementary file 5 — Supporting Information Figure 5. Inhibition of ATM signaling up-regulates SIRT1 mRNA levels. (A) Human umbilical vein endothelial cells (HUVEC) (n=3) were transfected with siRNA against ATM or control siRNA in low serum medium for 48h. SIRT1 mRNA levels were measured by RT-PCR. GAPDH was used as loading control. SIRT1 mRNA levels were significantly increased in ATM knock-down cells 48h post siRNA treatment. *p<0.05. [file stem0031-2813-sd5.tif]

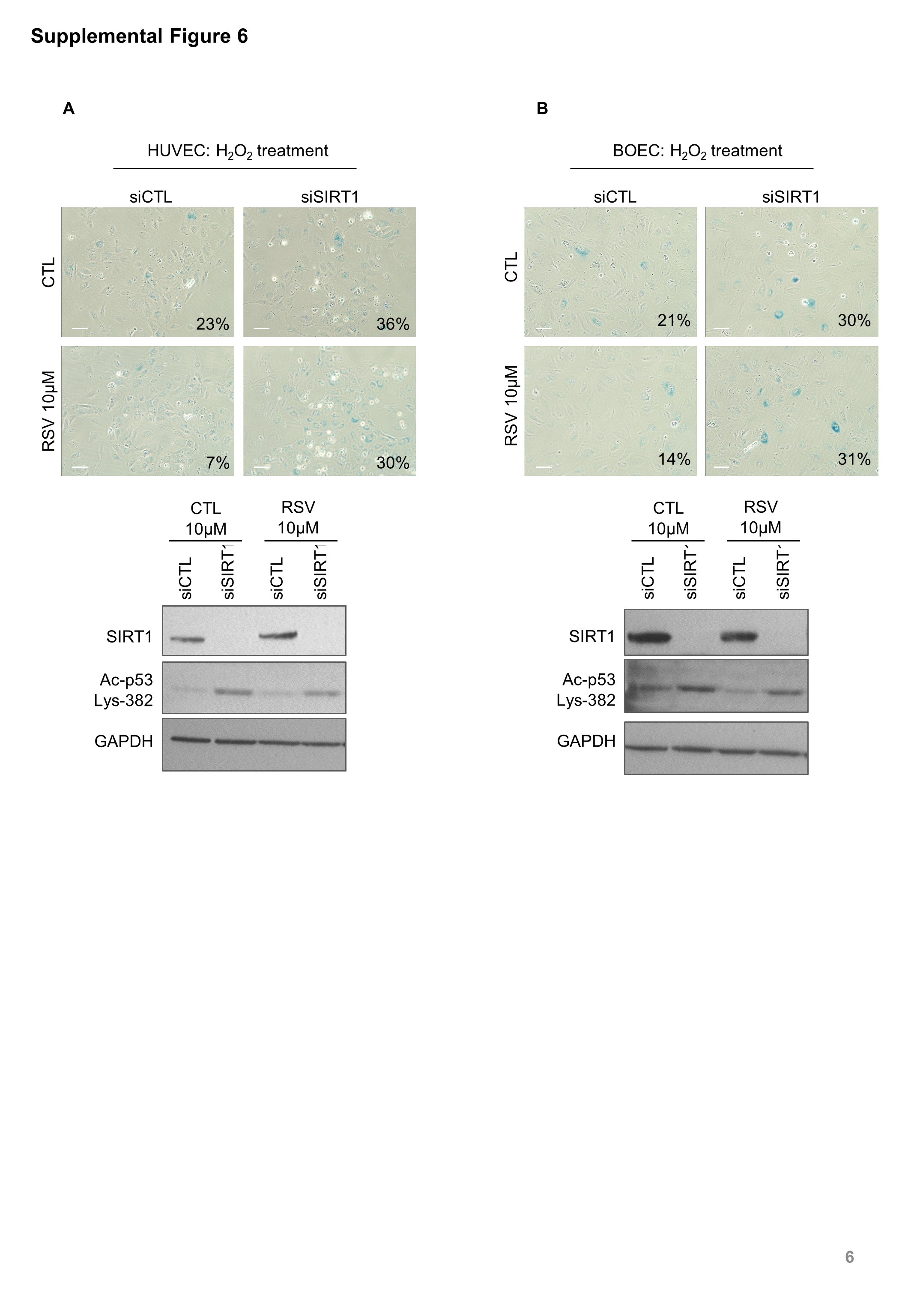

Supplement: Supplementary file 6 — Supporting Information Figure 6. Resveratrol inhibits premature senescence due to oxidative stress via SIRT1 activation. (A) Human umbilical vein endothelial cells (HUVEC) were transfected with siRNA against SIRT1 or control siRNA for 48h. After pretreatment with resveratrol (10μM) or control medium for one hour, cells were exposed to H2O2 (50μmol/L for 1 hour) to induce oxidative-stress premature senescence, then cultured for 48 additional hours with resveratrol or control media. Cellular senescence was assessed by SA-β-gal activity. Protein levels of SIRT1 and acetylation of p53 at Lys-382 were measured by Western blotting. GAPDH was used as loading control. Resveratrol treatment caused a reduction of SA-β-gal activity only in control siRNA treated cells. The effect was abolished in SIRT1-deficient HUVEC. Increased acetylation of p53 at Lys-382 was maintained in SIRT1-deficient cells compared to control cells after treatment with resveratrol. (B) Blood outgrowth endothelial cells (BOEC) from healthy non-smokers were treated as described for HUVEC. Resveratrol treatment inhibited senescence in control siRNA treated cells but not in SIRT1-deficient cells, which maintained increased acetylation of p53 (Lys-382). SA-β-gal activity is displayed as percentage of SA-β-gal positive cells relative to the total number of cells, at the right bottom corner of the images (scale bars 100μm). [file stem0031-2813-sd6.tif]

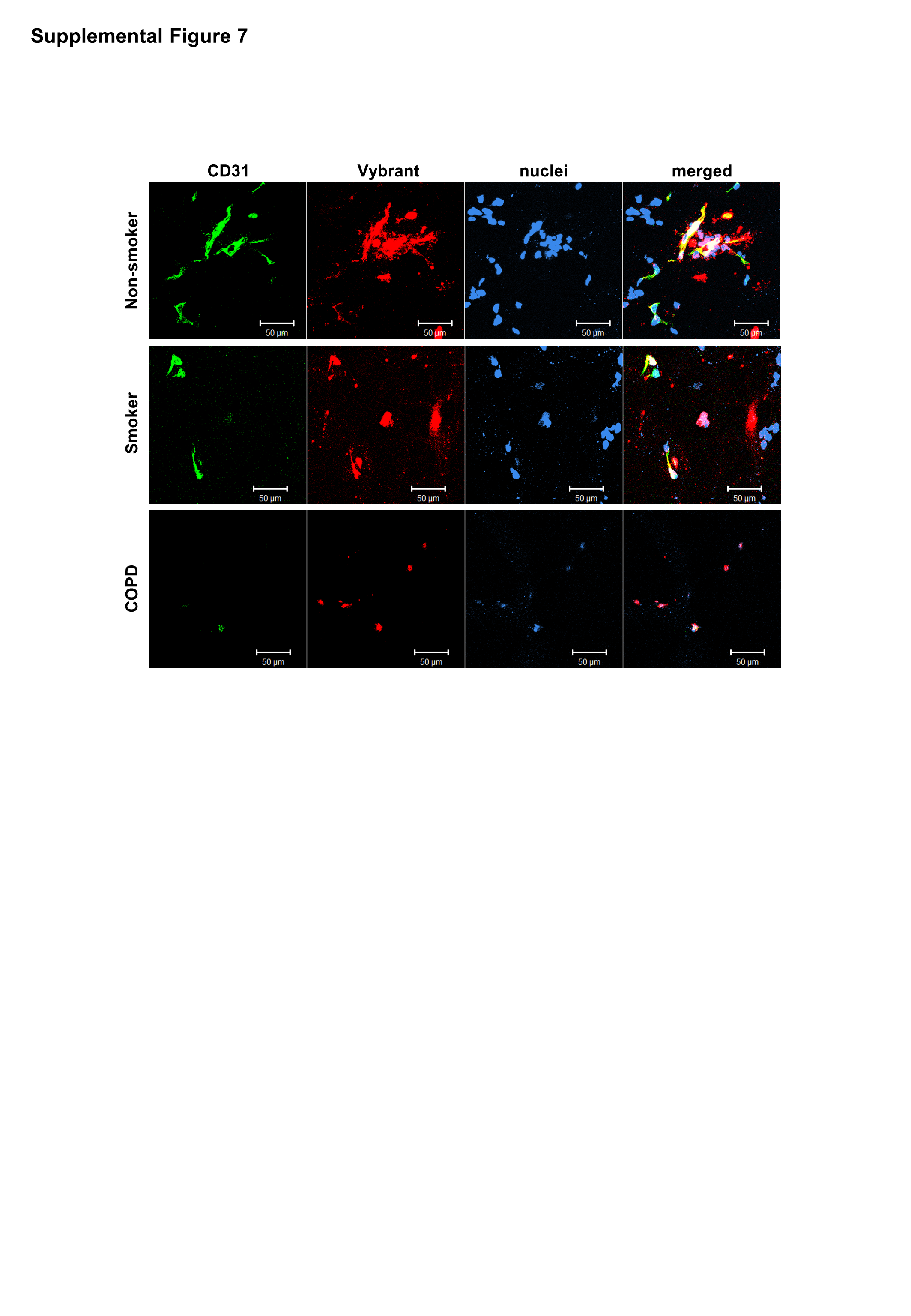

Supplement: Supplementary file 7 — Supporting Information Figure 7. BOEC from smokers and COPD patients show reduced angiogenic ability. BOEC from non-smokers (n=4), smokers (n=1) and COPD patients (n=3) were labeled with Dil-Vybrant cell solution, and were subcutaneously injected in SCID mice. Seven-day-old plugs were harvested, frozen, cryosectioned, and analysed by immunofluorescence confocal microscopy. Experiments were performed for each sample in duplicate. Immunofluorescent analysis for CD31 staining (green) revealed lining of the Vybrant positive cells and formation of capillary-like structures in the Matrigel plugs with BOEC from non-smokers but not in smokers and COPD samples (scale bars 50μm). [file stem0031-2813-sd7.tif]
